# The Association Between Periodontal Disease and Cardiovascular Disease: Insights From Imaging, Observational, and Genetic Data

**DOI:** 10.1016/j.jacadv.2024.101241

**Published:** 2024-08-31

**Authors:** Mihir M. Sanghvi, Julia Ramírez, Sucharitha Chadalavada, Nay Aung, Patricia B. Munroe, Nikolaos Donos, Steffen E. Petersen

**Affiliations:** a William Harvey Research Institute, Queen Mary University of London, London, United Kingdom; b Barts Heart Centre, Barts Health NHS Trust, London, United Kingdom; c Aragon Institute of Engineering Research, University of Zaragoza, Zaragoza, Spain; d Centro de Investigación Biomédica en Red–Biomedicina, Bioingeniería y Nanomedicina, Zaragoza, Spain; e Institute of Dentistry, Centre for Oral Clinical Research, Faculty of Medicine and Dentistry, Queen Mary University of London, London, United Kingdom

**Keywords:** cardiovascular magnetic resonance, cardiovascular risk factors, coronary artery disease, periodontal disease

## Abstract

**Background:**

Periodontal disease is the sixth most common disease worldwide and may be a contributory risk factor for cardiovascular disease (CVD).

**Objectives:**

This study utilizes noninvasive cardiac imaging and longitudinal and genetic data to characterize the association between periodontal disease and both cardiovascular magnetic resonance (CMR) imaging biomarkers of remodeling and incident coronary artery disease (CAD).

**Methods:**

From the UK Biobank, 481,915 individuals were included, 91,022 (18.9%) of whom had self-reported periodontal disease. For imaging analysis, 59,019 had paired CMR data. Multivariable linear regression models were constructed to examine the association of periodontal disease on CMR outcomes. The endpoints for the CMR analyses were left ventricle (LV) end-diastolic volume, LV ejection fraction, LV mass, LV mass:volume ratio, LV global longitudinal strain, and native T1 values. The relationship between periodontal disease and CVD was assessed using Cox proportional hazards regression models, with incident CAD as the endpoint. To examine the relationship of genetically determined periodontal disease on CAD, a genome-wide polygenic risk score was constructed.

**Results:**

Periodontal disease was associated with a significantly higher LV mass:volume ratio (effect size: 0.00233; 95% CI: 0.0006-0.004) and significantly lower T1 values (effect size: −0.86 ms; 95% CI: −1.63 to −0.09). Periodontal disease was independently associated with an increased hazard of incident CAD (HR: 1.09; 95% CI: 1.07-1.13) at a median follow-up time of 13.8 years. Each SD increase in the periodontal disease polygenic risk score was associated with increased odds of CAD (OR: 1.03; 95% CI: 1.02-1.05).

**Conclusions:**

Using an integrated approach across imaging, observational, and genomic data, periodontal disease is associated with biomarkers of subclinical remodeling as well as incident CAD. These findings highlight the potential importance of periodontal disease in the broader context of CVD prevention.

Despite advancements in the treatment and management of coronary artery disease (CAD), it remains a leading cause of mortality and morbidity, emphasizing the need to reduce the risk of developing CAD. While there is a vogue for identification of novel molecular biomarkers that confer heightened CAD risk, these are, in the main, limited by their lack of routine assessment and mitigation strategies.

Periodontal disease (gingivitis and periodontitis) is a condition that affects the dental supporting structures, including the gums, surrounding connective tissue, and alveolar bone. Gingivitis involves only inflammation of the gums, while periodontitis is a chronic inflammatory disease that is clinically expressed by the progressive destruction of the tooth-supporting apparatus (periodontium), with primary features being loss of clinical attachment and radiographic alveolar bone loss (leading to increased tooth mobility and tooth loss), and presence of periodontal pockets and bleeding on probing.^1^ Severe periodontal disease is estimated to affect more than 1 billion individuals globally and is the sixth most common disease worldwide.^2^^,^^3^ Several studies have suggested an association between periodontal disease and CAD, and importantly, from a public health perspective, prevention of periodontal disease is relatively straightforward and cost-effective by appropriate oral hygiene procedures such as daily brushing, interdental cleaning, and, where applicable, control of associated risk factors such as smoking.^4^, ^5^, ^6^

This study aims to provide an integrative exploration of periodontal disease as a risk factor for cardiovascular disease (CVD). Leveraging a sample size of close to half a million participants from the UK Biobank, we first examine the association of periodontal disease with changes in prognostically important cardiovascular magnetic resonance (CMR) phenotypes. CMR is the gold standard for the measurement of cardiac structural phenotypes and subclinical changes in morphological and functional parameters, which are known to be prognostic markers of cardiovascular morbidity and mortality.^7^, ^8^, ^9^ Secondly, we determine the risk of incident CAD in those with and without periodontal disease. Thirdly, we establish the risk of CAD from genetically determined periodontal disease through a genome-wide polygenic risk score.

## Methods

### Study cohort

The UK Biobank is a large population-based prospective cohort study of 500,000 individuals aged between 40 and 69 years at the time of initial recruitment between 2006 and 2010. It has collected information on health and lifestyle data, physical measurements, biological samples, and genotype. Incident health events are prospectively tracked through linkage to electronic health record data (hospital episode statistics [HES], death register).^10^

This study was conducted using UK Biobank access application 2,964 with adherence to the principles outlined in the Declaration of Helsinki concerning human subjects and tissues. It was covered by the general ethical approval for UK Biobank studies from the NHS National Research Ethics Service (June 17, 2011 [Ref 11/NW/0382]; extended on May 10, 2016 [Ref 16/NW/0274]) and on June 18, 2021 (Ref 21/NW/0157) with written informed consent obtained from all participants.

### CMR parameters

CMR examinations, as part of the UK Biobank imaging enhancement, have been performed from 2015 onward. The CMR protocol has been described in detail previously.^8^^,^^11^ Segmentation of the left ventricle (LV) to derive LV end-diastolic volume (EDV), LV mass, and LV ejection fraction (EF) were derived using automated machine learning algorithms as described previously.^12^ LV mass:volume ratio (MVR) was calculated by dividing the LV mass (g) by LV EDV (ml). LV global longitudinal strain (GLS) was derived using cvi42 software (prototype 5.13.7I, Circle Cardiovascular Imaging) using an automated, batch-processed feature tracking analysis. Global T1 values were measured in the short-axis view at the mid-ventricular level using a deep-learning algorithm as previously described, and measurements deemed to be of low quality (predicted Dice similarity coefficient score <0.7) were excluded.^13^ For all CMR parameters, values that lay 3∗IQR above the third quartile or below the first quartile were classed as outliers and excluded.

### Periodontal disease and coronary artery disease definitions

The presence of periodontal disease was defined as any participant indicating that they had “painful gums,” “bleeding gums,” and/or “loose teeth” as part of the UK Biobank health questionnaire completed at the baseline assessment visit (UK Biobank data-field 6149). The utilization of self-reported measures of periodontal disease has been validated against clinical definitions of periodontal disease in a range of populations.^14^^,^^15^

CAD was defined using International Classification of Diseases (ICD)-10 diagnostic codes (I20, I21, I22, I23, I24, I25, and their subclasses). ICD-10 codes are incorporated into the UK Biobank dataset via linkage with HES, a comprehensive database of all admissions, outpatient appointments, and emergency department visits to NHS hospitals. If a UK Biobank participant receives hospital care, a health care provider assigns ICD-10 codes to describe diagnoses related to that care. Incident CAD was defined as any code logged at a timepoint postdating a participant’s baseline assessment visit.

### Observational analysis

Baseline data are presented in categorized fashion with participants grouped by presence of periodontal disease or not. Descriptive statistics were computed and presented as mean ± SD for continuous variables and percentages for categorical variables. Continuous variables were compared using independent samples t-tests. For categorical variables, chi-square tests were used.

To examine the association between presence of periodontal disease and CMR parameters, multivariable linear regression models were fitted for each dependent variable (LV EDV, LV EF, LV mass, LV MVR, LV GLS, and global native T1). Covariates included age, sex, ethnicity, systolic and diastolic blood pressure, body mass index, smoking status, HbA1c, Townsend deprivation index, low-density lipoprotein (LDL) cholesterol levels, and C-reactive protein (CRP). Individuals with known CAD were excluded from the analyses. To note, the Townsend deprivation index is a UK-specific composite measure of socioeconomic deprivation. It is calculated using an individual’s postcode and census data regarding unemployment, nonhome ownership, noncar ownership, and household overcrowding to generate a single deprivation index score.^16^ To verify assumptions of the linear regression models, linearity was assessed by plotting residuals against fitted values, residual independence was assessed using the Durbin-Watson test, and normality was visually assessed using Q-Q plots.

The relationship between periodontal disease and the incidence of CAD was assessed using Cox proportional hazards regression models, providing HRs as measures of risk. The outcome variable was time to incident CAD, with the presence or absence of periodontal disease serving as the main predictor. The included timeframe was date of initial recruitment until the occurrence of the first CAD event, or until a censor date of 8th August 2022 for those who did not experience an event. The Cox models were again adjusted for the following covariates: age, sex, ethnicity, systolic and diastolic blood pressure, body mass index, smoking status, HbA1c, Townsend deprivation index, LDL cholesterol levels, and CRP. Survival curves were generated to graphically represent the results of the Cox proportional hazards regression analysis using the “ggadjustedcurves” function in the survminer R package. Plots of Schoenfeld residuals were visually assessed to assess for model violations. In addition to this primary analysis for risk of incident CAD, noncardiovascular death was considered as a competing risk. To account for this, a Fine-Gray subdistribution hazard model was constructed to estimate the association of periodontal disease and incident CAD in the presence of noncardiovascular death as a competing risk using the cmprsk R package.

As a sensitivity analysis, to examine whether periodontal disease is acting as a mediating variable between socioeconomic status as determined by the Townsend deprivation index score and cardiovascular parameters and outcomes, analyses were repeated with the Townsend score excluded from the covariates.

All observational analyses were conducted in R version 4.1.1.^17^

### Polygenic risk score derivation

In order to generate the polygenic risk score (PRS) for periodontal disease, a genome-wide association study (GWAS) was first performed.

Participants in UK Biobank were genotyped using the UK BiLEVE and UK Biobank axiom array. Preimputation quality control, phasing, and imputation were performed at the Wellcome Trust Centre for Human Genetics prior to data release. Analysis used the 2018 (v3) imputed data release including imputation to both Haplotype Reference Consortium and UK10K/1,000 genomes combined panel.^18^

Participant-level quality control involved restricting analysis to participants of European ancestry (identified as the largest cluster formed after k-means clustering on genetic principal components) as well as standard exclusions for mismatch between reported and genetic sex, possible sex chromosome aneuploidy, outliers in heterozygosity, and missing rates. Participants who met the definition of periodontal disease as stated above were coded as cases for the genome-wide analysis. The GWAS included 81,344 cases and 358,904 controls. Association analysis was performed using REGENIE v2.2.4 via the 2-step procedure.^19^ In brief, the first step fits a whole genome regression model for trait (periodontal disease) prediction based on genetic data using the leave one chromosome out scheme. Genotyped variants were filtered according to a minor allele frequency of >1%, Hardy-Weinberg equilibrium test *P* > 10^-6^ and <0.15% missingness. The leave one chromosome out predictions are used as offsets in step 2, where variant association analyses across the imputed chromosomal data are performed using approximate Firth regression when the *P* value from the standard logistic regression is <0.01. Imputation score (INFO) >0.3 was used, and the association models included the following covariates: age, age,^2^ sex, genotyping array, and the first 10 genetic principal components. Univariate linkage-disequilibrium score regression was used to estimate genome-wide single nucleotide polymorphism-based heritability.

A PRS for periodontal disease was generated using PRS-CS.^20^ PRS-CS utilizes a Bayesian framework to model linkage disequilibrium from an external reference set and a continuous shrinkage prior to single nucleotide polymorphism effect size. The European panel from the 1000 Genomes project was used as a reference, and the PRS-CS *auto* option was employed to allow the software to learn the continuous shrinkage prior to the data. The resulting variant weights were then used to calculate the PRS using PLINK v1.9. To examine the genetically determined risk of periodontal disease, we examined the association between the PRS and CAD in both a continuous fashion and comparing individuals in the top and bottom PRS quintiles in logistic regression models using the same covariates as in the observational model as well as the first 10 genetic principal components. The PRS was scaled to a mean of 0 and SD of 1 resulting in the coefficients being outputted as log-odds; these were then exponentiated to produce odds ratios.

## Results

In this study, 481,915 individuals for whom oral health data was available were included. Periodontal disease occurred in 18.9% (n = 91,022) individuals. Baseline characteristics for the study population, divided by those with and without periodontal disease, are presented in Table 1. Those in the periodontal disease group were younger (54.7 vs 56.6 years), were more likely to be of self-reported non-White ethnicity, had a higher body mass index (27.7 vs 27.2 kg/m^2^), were more likely to be current or previous smokers (47.4% vs 44.2%), had a higher deprivation index, and had higher levels of CRP (2.7 vs 2.5 mg/L).

**Table 1:** Baseline Demographics

	Periodontal Disease (n = 91,022, 18.9%)	No Periodontal Disease (n = 390,893, 81.1%)
Age, y	54.9 ± 7.9	56.6 ± 8.1^a^
Female	60.1	54.4^a^
Ethnicity		
Asian	4.59	3.70^a^
Black	0.75	0.52^a^
Chinese	0.51	0.28^a^
Mixed	4.61	3.56^a^
Other	1.25	0.83^a^
White	88.3	91.1^a^
BMI, kg/m^2^	27.7 ± 5.1	27.2 ± 4.7^a^
Systolic BP, mm Hg	138 ± 19	140 ± 20^a^
Diastolic BP, mm Hg	82 ± 11	82 ± 11
Ever smokers	47.4	44.2^a^
HbA1c	36.0 ± 7.0	35.9 ± 6.4^a^
Townsend deprivation index	19.1 ± 14.7	17.2 ± 13.7^a^
LDL cholesterol	3.58 ± 0.86	3.59 ± 0.86^a^
CRP	2.74 ± 4.40	2.53 ± 4.28^a^

Values are mean ± SD or %. Continuous variables are compared using independent samples t-tests. For categorical variables, chi-square tests are used.

BMI = body mass index; BP = blood pressure; CRP = C-reactive protein; LDL = low-density lipoprotein.

a *P* ≤ 0.05.

The association of the presence of periodontal disease on prognostically important left ventricular CMR parameters in fully adjusted models are presented in Table 2. The following number of participants had paired self-reported periodontal disease and CMR data and were included in the analyses: 59,019 individuals for LV EDV, EF, mass, and MVR; 44,374 for LV GLS; 41,771 for global native T1. The mean time between oral health data collection and CMR examination was 8.8 years. Periodontal disease was associated with a significantly higher LV MVR (effect size: 0.00233; 95% CI: 0.0006–0.004; *P* = 0.0062) and significantly lower LV native global T1 value (effect size: −0.86 ms; 95% CI: −1.63 to −0.09; *P* = 0.0285). There were no significant differences observed for LV EDV, LV mass, or LV EF.

**Table 2:** Association of Periodontal Disease on Prognostically Important CMR Phenotypes

	Effect Size	95% CI	*P* Value
LV EDV (mL)	−0.42	−0.96 to 0.127	0.129
LV EF (%)	0.01	−0.115 to 0.135	0.918
LV mass (g)	0.07	−0.226 to 0.370	0.635
LV MVR	0.0023	0.0006–0.004	0.006^a^
LV GLS (%)	0.016	−0.033 to 0.065	0.531
Native T1 value (ms)	−0.860	−1.63 to −0.09	0.028^a^

Effect sizes are for presence of periodontal disease in regression models with the CMR parameter as the outcome and adjusted for age, sex, ethnicity, systolic and diastolic blood pressure, body mass index, smoking status, HbA1c, Townsend deprivation index, LDL cholesterol level, and C-reactive protein. Two-sided *P* values were computed from the multivariate linear regression models.

Fifty-nine thousand nineteen individuals for LV EDV, EF, mass, and MVR; 44,374 for LV GLS; 41,771 for global native T1.

CMR = cardiovascular magnetic resonance; EDV = end-diastolic volume; EF = ejection fraction; GLS = global longitudinal strain; LV = left ventricular; MVR = mass:volume ratio.

a Denotes a significant result.

**Figure 1:**
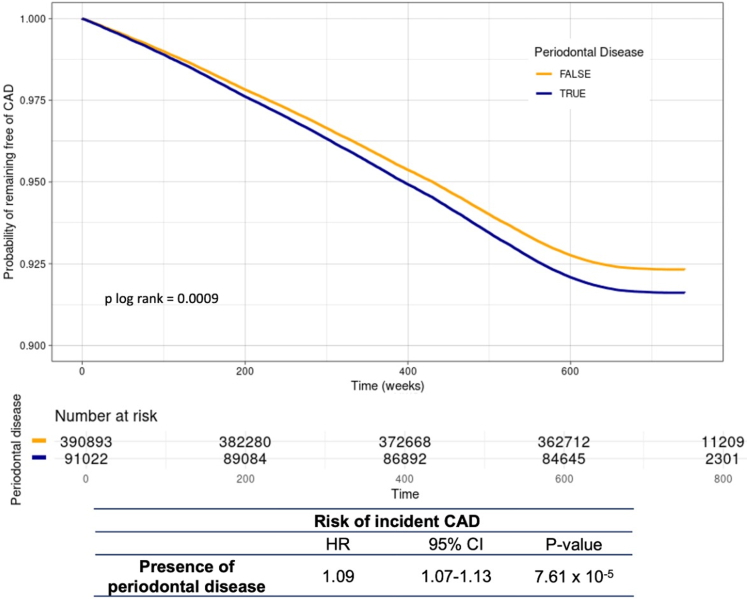
**The Association of Self-Reported Periodontal Disease and Incident CAD** Adjusted survival curves denoting risk of incident CAD from Cox proportional hazards model by presence of periodontal disease. Blue line represents those with self-reported periodontal disease, and yellow line represents those without. A total of 9,022 individuals are in the periodontal disease group, and 390,893 individuals are in the no periodontal disease group. Two-sided *P* value calculated using Cox proportional hazard models for association between periodontal disease and incident CAD and *P* value from log rank test used to compare distributions between the periodontal disease and no periodontal disease groups. CAD = coronary artery disease.

In total, there were 30,035 CAD events in the no periodontal disease group and 6,759 events in the periodontal disease group. The median follow-up time was 13.8 years (interquartile range: = 13-14.5 years). The association between periodontal disease and risk of incident CAD in fully adjusted Cox proportional hazard model is presented in Figure 1. The presence of periodontal disease was independently associated with a significantly increased hazard of incident CAD (HR: 1.09; 95% CI: 1.07–1.13; *P* = 7.61 × 10^-5^; *P* log-rank = 0.0009). This association between periodontal disease and incident CAD remained significant when accounting for noncardiovascular death as a competing risk (subdistribution HR: 1.07; 95% CI: 1.04–1.10; *P* = 3.5 × 10^-5^).

In sensitivity analyses, there was no difference in effect estimates noted when the Townsend deprivation index was removed as a covariate from the analyses.

**Figure 2:**
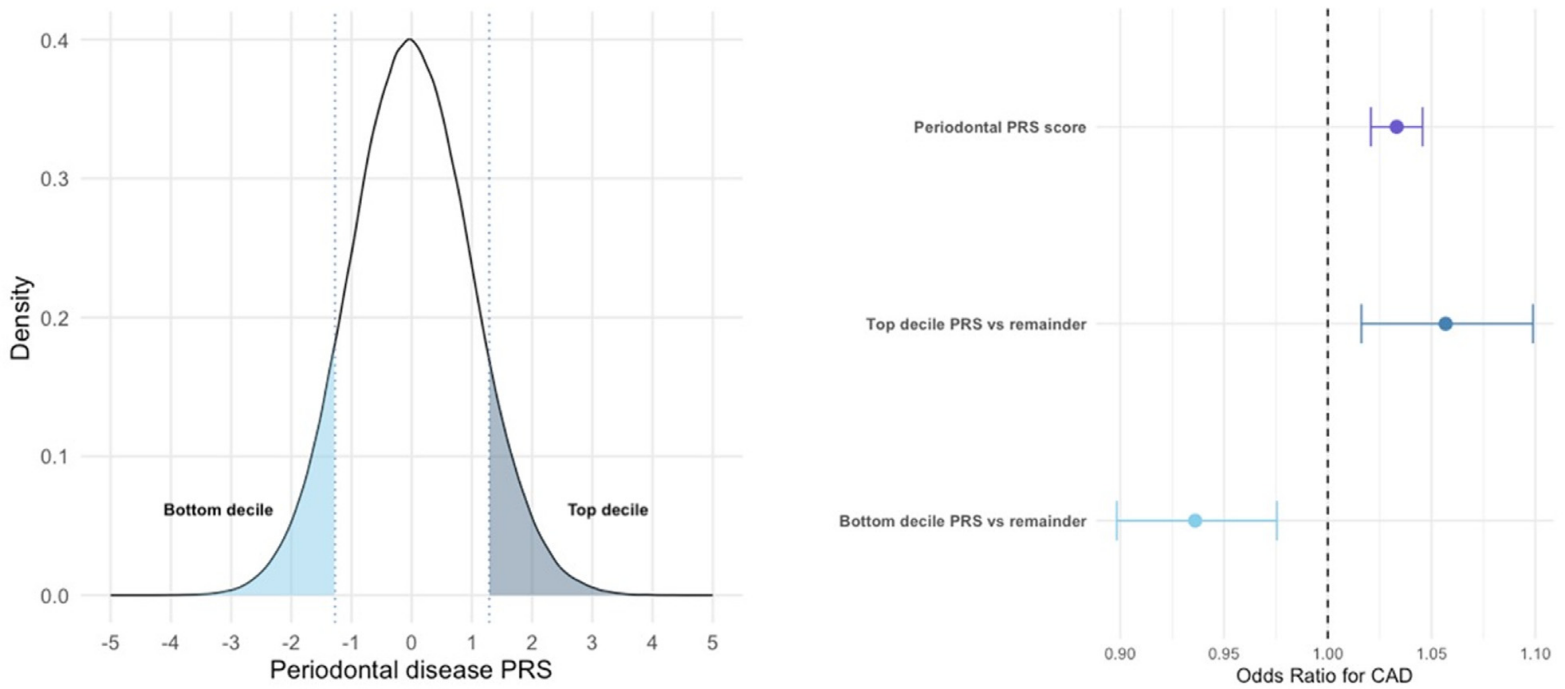
**The Association of Genetically-Determined Periodontal Disease and CAD** PRS for periodontal disease and risk of CAD stratified by entire cohort, top decile for PRS (dark blue shading) and bottom decile (light blue shading) for PRS. In the analysis, 467,350 individuals were included. Circles represent ORs, and error bars represent 95% CIs. CAD = coronary artery disease.

In the genetic analyses, 467,350 European individuals were included. There was evidence for association at genome-wide significance (*P* < 5.0 × 10^-8^) for a single, novel risk locus, *CCDC91*. The lead signal was rs7316831, a common intronic variant within *CCDC91* (allele frequency for A allele = 0.40; *P* = 4.35 × 10^-9^; OR for periodontal disease = 0.96). The heritability estimate was 2.13% (standard error = 0.0015) in keeping with previous studies.^21^ The genomic control factor (λ_GC_) was 1.171 with an linkage disequilibrium score regression intercept of 1.01 indicating modest inflation reflective of the likely polygenic nature of the periodontal disease trait. The association of genetically determined periodontal disease, as represented by a PRS, with CAD is presented in Figure 2. In a fully adjusted model, each SD increase in the periodontal disease PRS was associated with a 3.3% increased odds of CAD (OR: 1.033; 95% CI: 1.021–1.046; *P* < 0.00001). Individuals with a periodontal PRS in the top decile were at significantly increased risk of CAD compared to the remainder of the cohort (odds ratio: 1.057; 95% CI: 1.016–1.099; *P* = 0.0056). In contrast, those in the bottom decile for periodontal disease PRS were at significantly lower risk of CAD compared to the rest of the cohort (OR: 0.936; 95% CI: 0.898–0.976; *P* = 0.0017).

## Discussion

This large-scale, population-based study of self-reported periodontal disease demonstrated the following: firstly, periodontal disease is significantly associated with prognostic LV parameters as derived by CMR, namely LV MVR and global T1. Secondly, at a median duration of 13.5 years, there is a 9% increased risk of incident CAD in those with self-reported periodontal disease at baseline. Thirdly, a similar, positive association was observed for genetically determined periodontal disease as derived from a genome-wide PRS (Central Illustration).

**Central Illustration:**
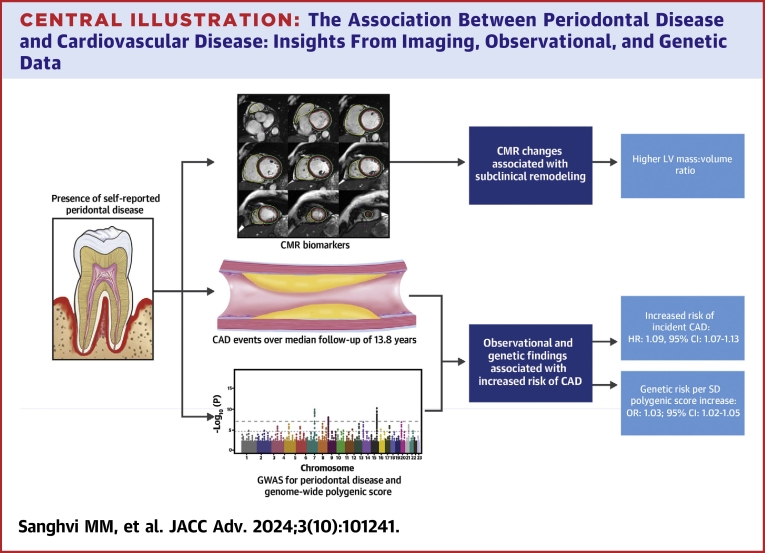
**The Association Between Periodontal Disease and Cardiovascular Disease: Insights From Imaging, Observational, and Genetic Data** Self-reported periodontal disease is associated with cardiac remodeling and incident coronary artery disease. For self-reported periodontal disease in a large-scale population-based study, there is evidence of association with markers of adverse, subclinical remodeling as measured by cardiovascular magnetic resonance imaging and with incident coronary artery disease on longitudinal follow-up (median 13.8 years). For genetically determined risk of periodontal disease as assessed by a polygenic risk score, there is association with significantly increased risk of coronary artery disease. CAD = coronary artery disease; CMR = cardiovascular magnetic resonance; GWAS = genome-wide association study.

This is the largest study to examine the effect of periodontal disease on CMR parameters. The finding of periodontal disease being associated with higher LV MVR is novel. LV MVR is known to be powerful biomarker for subsequent adverse cardiovascular events and is indicative of concentric remodeling.^7^ A previous study using M-mode echocardiography in patients with hypertension showed LV mass to be an independent predictor of severe periodontal disease, but a more contemporary echocardiographic study in healthy subjects did not show any associations between periodontal disease and alterations in structure and function.^22^^,^^23^ While these studies have been limited by their sample size (circa 100 subjects) and the relative imprecision in characterizing hypertrophy compared to CMR, there has been considerable interest in investigating mechanistic links between periodontal disease and myocardial hypertrophy. In murine transverse aortic constriction models, it has been shown that bacteria associated with periodontal disease promote development of LV hypertrophy. The presence of periodontal pathogens promotes an inflammatory state leading to oxidative stress in the endothelium of the coronary microvasculature. The resultant reduction in nitric oxide bioavailability leads to reduced protein kinase G activity in myocytes and induces hypertrophy.^24^ It is also notable that periodontal disease has been genetically and experimentally linked to hypertension, a well-known driver of hypertrophy, indicating the possibility of periodontal disease promoting adverse remodeling both directly and indirectly.^25^

The paradigm of periodontal disease as an inflammatory disorder in response to bacteria (most usually gram-negative) is well-established.^26^ In this regard, examining native T1 mapping values—albeit limited to a single, mid-ventricular short-axis slice—at population scale and examining their association with periodontal disease was attractive. T1 mapping refers to creation of a map with pixel-wise illustrations of absolute T1 relaxation times. These native T1 values are a composite of signals from myocytes, other cells, and the extracellular space (both the interstitium and intravascular space). As such, T1 mapping permits the detection of diffuse myocardial structural alterations that cannot be determined via other noninvasive means. They are not specific, however, with alterations in native T1 values a result of disease processes affecting all of these compartments. Native T1 values have been shown to be elevated not only in myocardial inflammatory states such as myocarditis but also in systemic inflammatory disorders such as rheumatoid arthritis and systemic sclerosis.^27^^,^^28^

The finding of self-reported periodontal disease being associated with lower native T1 values, therefore, may be considered surprising. Until now, native T1 has been studied predominantly as a clinical measurement in patients with overt disease rather than as a subclinical biomarker at population scale. In the fully adjusted model, it was notable that several cardiovascular risk factors such as body mass index, systolic blood pressure, and LDL-cholesterol were also significantly associated with lower T1 values—also shown by others^9^—in contrast to HbA1c levels and serum CRP, which were independently associated with higher T1 values. It would appear that modifiable risk factors for CVD significantly associate with native T1, although with differing effect estimate directions. The mechanisms for the association of these traditional cardiovascular risk factors, such as blood pressure and LDL cholesterol, as well as periodontal disease are difficult to explain; one would expect higher native T1 values both in the context of inflammation and/or ischemia. It is important to consider whether this represents a false positive result or whether the self-reported periodontal disease phenotype heterogeneity results in native T1 not being an effective biomarker as an endpoint in this context. The only previous study to examine the relationship between periodontal disease and T1 parameters was a study from the Multi-Ethnic Study of Atherosclerosis (MESA).^29^ At a follow-up interval of 10 years, they showed no significant differences in T1 values and self-reported periodontal disease. In men only, they showed periodontal disease to be associated with a higher extracellular volume fraction, which is a T1-derived parameter that can only be determined following administration of intravenous contrast (not performed in the UK Biobank) and is a marker of collagen accumulation.

We report a 9% increased risk of incident CAD in those with periodontal disease. This association is consistent with the previous literature. As examples, the PAROKRANK (Periodontitis and its Relationship to Coronary Artery Disease) study demonstrated a 28% increased risk of myocardial infarction in those with periodontal disease.^30^ Similarly, from the Danish National Registry, using a cohort of 17,691 periodontal disease cases with approximately 83,000 controls demonstrated a 16% increased risk of myocardial infarction in those with periodontitis.^31^ The greater effects observed in these studies may be due to periodontitis, that is, the more severe spectrum of periodontal disease, being the exposure of interest as well as performing objective grading/diagnosis via clinical examination rather than self-report as in this study.

We sought to characterize genetically determined risk of periodontal disease through a genome-wide PRS. The periodontal PRS was significantly associated with increased risk of CAD with those in the top and bottom deciles for periodontal disease PRS demonstrating opposite effect size directionality for incident CAD. While a GWAS has previously been performed for periodontal disease in the UK Biobank, it was felt that the approach adopted by the authors in their definition of periodontal “cases” was not physiological: including mouth ulcers (aphthous ulcers are not strictly associated with periodontal disease) in their definition of periodontal disease as an example.^21^ The PRS derived here used the same definition of periodontal disease as in the observational analysis.

While Mendelian randomization approaches offer the opportunity to determine “causality,” we opted not to pursue this approach due to weakness of a genetic instrument for periodontal disease. Given this study focuses on an integrated approach to periodontal disease as a risk factor for CAD, we chose to employ a genome-wide PRS strategy (ie, a very large number of contributory variants) rather than a variant-specific PRS due to this having a better application in the risk prediction and stratification of an exposure rather than in assessing causaility.^32^ While our model exploring the association between the periodontal PRS and incident CAD was adjusted for potential confounders, it is possible that the relationship may be explained by pleiotropy. This may be through periodontal disease and CAD sharing an overlapping genetic architecture meaning that there are shared genetic factors that would predispose an individual to both conditions, although a lookup of reported periodontal disease-associated loci in a large GWAS for CAD did not reveal any shared significant variants.^33^^,^^34^ Similarly, the PRS may be capturing indirect effects whereby the effect on CAD may be via pathways not directly related to periodontal disease.

This study benefits from several advantages. Firstly, it is the largest investigation of the association of prognostically important remodeling parameters derived from CMR. Secondly, we extend existing observations of the association between periodontal disease and CAD in a cohort of nearly half a million individuals. Interventions such as tooth brushing and interdental cleaning, as well as improving the technique of these oral hygiene procedures are low-cost and can be instituted from a young age. In the UK, extraction of decayed teeth is the most common reason for children to undergo general anesthetic and CVD accounts for £7.4 billion of expenditure in direct costs to the UK health system each year.^35^ This underscores the importance of tackling periodontal disease at a public health scale.

There are several limitations to this study. Firstly, this is an observational study and, therefore, susceptible to confounding; while the statistical analyses performed have adjusted for a range of covariates, residual confounding cannot be ruled out. Secondly, it is known that the UK Biobank study population is affected by a “healthy volunteer” selection bias with regards to the cohort being generally healthier than the overall population. Furthermore, due to considerations of sample size, the genetic analyses were restricted to individuals of European ancestry, limiting the generalizability of the findings to other ancestries. Thirdly, periodontal disease was defined from self-reported proxy measures rather than via clinical examination. Oral clinical examination did not form part of the UK Biobank protocol. Due to the majority of dental care in the UK taking place in the community setting or in the private sector, we were also unable to rely upon clinical coding from HES due to the paucity of data. It is acknowledged that these self-reported data are particularly vulnerable to bias, particularly measurement error, which in turn can lead to individuals being “misclassified” in terms of periodontal disease status but also information bias, whereby the data are affected by misreporting or inaccuracies in recall.^36^ The lack of clinical examination and linked dental records makes the issue of misclassification of particular concern. While the use of questionnaire responses has been validated as being an acceptable mechanism for population surveillance, it is noted that the specificity of this approach is higher than the sensitivity, thereby likely to generate an overall underestimate of the true prevalence due to the number of false negatives. Fourthly, as well as the potential for misclassification, it is recognized that periodontal disease is a heterogeneous condition and therefore represents a broad clinical spectrum. Self-reported metrics are unable to account for this variability and therefore will affect the strength of associations with outcomes.

## Conclusions

Using an integrated approach across imaging, observational, and genetic data, this study provides evidence that periodontal disease is associated with biomarkers of adverse subclinical remodeling as well as incident CAD. These findings highlight the potential importance of periodontal disease in the broader context of CVD prevention.

Perspectives **COMPETENCY IN PATIENT CARE AND PROCEDURAL SKILLS:** Periodontal disease is a prevalent condition and may be a potential risk factor for coronary artery disease. Incorporating questions around oral health during clinical consultations can provide opportunities for detection and treatment of periodontal disease via an onward visit to a dental professional.

**COMPETENCY IN MEDICAL KNOWLEDGE:** Periodontal disease is associated with features of subclinical adverse remodeling as detected by cardiovascular magnetic resonance imaging.

**TRANSLATIONAL OUTLOOK:** This study seeks to highlight the potential role of periodontal disease in development of CAD through a multimodal approach. Given the range of preventative and therapeutic measures available in the management of periodontal disease, we hope that this study will ignite interest in performing further evaluation of periodontal disease intervention on incidence and progression of CAD.

## Funding support and author disclosures

Dr Sanghvi recognizes his British Heart Foundation’s (BHF) Clinical Research Training Fellowship (FS/CRTF/22/24353). Dr Chadalavada is supported by the European Union's 10.13039/100010661Horizon 2020 research and innovation program under grant agreement no. 825903 (euCanSHare project). Dr Aung recognizes his 10.13039/501100000265Medical Research Council (MRC) Clinician-Scientist Award (MR/X020924/1). Drs Munroe and Petersen acknowledge the support of the National Institute for Health and Care Research Barts Biomedical Research Centre (NIHR203330), a delivery partnership of Barts Health NHS Trust, Queen Mary University of London, St George’s University Hospitals NHS Foundation Trust, and St George’s University of London. Dr Petersen acknowledges the British Heart Foundation for funding the manual analysis to create a cardiovascular magnetic resonance imaging reference standard for the UK Biobank imaging resource in 5,000 CMR scans (PG/14/89/31194). Barts Charity (G-002346) contributed to fees required to access UK Biobank data [access application #2964]. All other authors have reported that they have no relationships relevant to the contents of this paper to disclose.
